# Establishing rabbit critical-size bone defects to evaluate the bone-regeneration potential of porous calcium phosphate ceramics

**DOI:** 10.3389/fbioe.2024.1524133

**Published:** 2025-01-29

**Authors:** Wei Lei, Yan Wu, Hao Yuan, Ping He, Jingqi Wu, Jingrong Chen, Yuxiao Liu, Hongmei Zhang, Joost D. de Bruijn, Xuerong Xiang, Ping Ji, Huipin Yuan, Mingzheng Li

**Affiliations:** ^1^ Chongqing Key Laboratory of Oral Diseases, Chongqing Municipal Key Laboratory of Oral Biomedical Engineering of Higher Education, Stomatological Hospital of Chongqing Medical University, Chongqing, China; ^2^ Department of Stomatology, Affiliated Hospital of North Sichuan Medical College, Nanchong, China; ^3^ Huipin Yuan’s Lab, Chengdu, China; ^4^ Kuros Biosciences BV, Bilthoven, Netherlands

**Keywords:** critical-size bone defect, bone regeneration, bone substitutes, calcium phosphate ceramic, submicron surface topography

## Abstract

Critical-size bone defects (CSDs), which are those that do not self-repair in a given period, are essential for evaluating bone-regeneration strategies. We established CSDs models in the rabbit cranium and ulna, and the bone-regeneration capacities of porous calcium phosphate (CaP) ceramics were assessed. A 12.6-mm cranial defect was confirmed as a CSDs after 12 weeks, with submicron surface-structured biphasic calcium-phosphate (BCP) implants [consisting of 20% hydroxyapatite and 80% tricalcium phosphate (TCP)] demonstrating significantly higher bone formation (32.2% ± 10.6%) than micron surface-structured TCP (TCP-B) implants (17.8% ± 4.6%, *p* = 0.0121). Ulna defects (15.0 mm in length) failed to heal spontaneously within 24 weeks when the periosteum was removed from both the ulna and radius, and the radius was covered with an expanded polytetrafluoroethylene (ePTFE) membrane. No bone bridging (i.e., union) was observed in the BCP implants at 12 weeks, whereas 80% of BCP implants (four out of five) achieved union by 24 weeks. Furthermore, the bone area within the available space of BCP implants increased significantly from 19.3% ± 7.3% at 12 weeks to 37.7% ± 8.5% at 24 weeks (*p* = 0.0063), accompanied by significant BCP resorption (14.8% at 12 weeks and 30.2% at 24 weeks). This study offers two rabbit CSDs models for evaluating bone-regeneration strategies (including bone substitution), and the overall data obtained in the current study indicate the possibility of repairing CSDs with CaP ceramics demonstrating improved bone-forming ability given adequate implantation time.

## Graphical Abstract

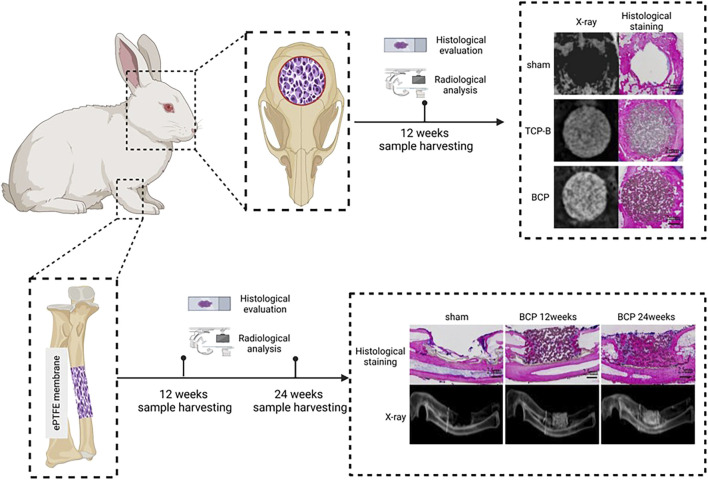

## Highlights

• This study successfully established two reliable critical-size bone-defect models in the rabbit calvaria and ulna, offering robust platforms for the evaluation of bone-regeneration potential of bone-substitute materials.
• Biphasic CaP ceramics with submicron-scale surface topography demonstrated promising potential in CSDs repair.

## 1 Introduction

In addition to hosting the hematopoietic system (Lucas, 2021), bone forms the skeletal system, which protects important organs (such as the brain, heart, and lung) and permits locomotion (Cowan et al., 2024). Bone damage causes pain (e.g., lower back pain in the spine) and bodily deformation, significantly impacting daily life (Global et al., 2021).

Bone damage can be caused by trauma, inflammation, tumors, and various congenital diseases. While minor bone injuries can self-heal, the large bone defects have a long healing time and can amount to CSDs that cannot self-heal in a given time (Schemitsch, 2017), potentially necessitating bone grafting for bone reconstruction (Bauer and Muschler, 2000).

Various bone-grafting materials are available for clinical use to repair bone defects (Bauer and Muschler, 2000). The use of autologous bone is the gold standard (Bauer and Muschler, 2000; Pape et al., 2010), but it is limited in terms of the amount of available bone, and it also necessitates a second surgery, which increases the financial burden and imposes more physical trauma on patients (Bauer and Muschler, 2000; Pape et al., 2010; Schmidt, 2021). Moreover, using autologous bone in functional bone repair is not always successful in clinics because it can be rapidly resorbed before complete bone healing occurs (Yang et al., 2020). Moreover, allogeneic bone from donors is another option (Bauer and Muschler, 2000), but it has several disadvantages, including potential immunological reactions, disease transmission, and inferior bone-forming capacity, compared with autologous bone (Bauer and Muschler, 2000). Synthetic bone substitutes are, therefore, attractive because such bone-grafting materials can be made available in large quantities, avoid an antigenic response, and have no ethical restrictions (Fernandez de Grado et al., 2018). However, the inferior bone-forming ability of synthetic bone-grafting materials limits their use in bone regeneration (Bauer and Muschler, 2000; Fernandez de Grado et al., 2018).

Being the primary inorganic component of bone, CaP materials are considered promising candidates for bone substitutes (Hou et al., 2022). Among them, hydroxyapatite (HA) ceramics (Fiume et al., 2021), tricalcium phosphate (TCP) ceramics (Bohner et al., 2020), and BCP ceramics are the most investigated ceramics (Bouler et al., 2017). Although the osteogenic potential of CaP ceramics is limited, the bone-formation ability of CaP ceramics varies with their physicochemical properties (Samavedi et al., 2013), shedding light on further improving the potential of CaP ceramics with respect to their bone-forming ability for bone regeneration.

The biological evaluation of bone substitutes (including CaP ceramics) is crucial in further optimizing bone substitutes. Culturing osteogenic cells (e.g., bone-marrow stromal cells and osteoblast-like cells) on bone substitutes and observing their cell proliferation and osteogenic differentiation have revealed clues about the bone-forming ability of the bone substitutes subjected to testing (Müller et al., 2008), while other factors affecting *in vivo* bone formation (e.g., inflammatory response and angiogenesis) may not be simultaneously investigated *in vitro* (Maruyama et al., 2020; Stegen et al., 2015). From no bone to bone, the ectopic implantation (e.g., in muscle or under the skin) of bone substitutes has generated the most reliable evidence to show the bone-forming ability of bone substitutes (Barradas et al., 2011; Veronesi et al., 2020). However, the functionality of bone substitutes for bone regeneration has ultimately to be validated in orthopedic sites since the environments hosting bone formation have been found to vary between ectopic and orthopedic sites [e.g., the sources of osteogenic cells (Albrektsson and Johansson, 2001) and mechanical loadings (Watanabe-Takano et al., 2021)].

Given that bone can self-repair small-scale damage, it is essential to exclude the influence of the bone’s intrinsic regenerative capacity when evaluating or comparing the bone-regeneration potential of bone substitutes in orthopedic implants. CSDs represent an essential platform for such investigations, providing a reliable framework for assessing the bone-regeneration potential of different materials under standardized conditions (Brunello et al., 2020). To further account for variables including the influence of the surrounding bone (Albrektsson and Johansson, 2001), the periosteum (Jeyaraman et al., 2021), and animal age (Hirata et al., 2022) on bone formation in bone defects, this study established CSD models in the rabbit cranium and ulna. These models may offer a robust and reproducible method to investigate the performance of bone substitutes or other bone-regeneration strategies (Jing et al., 2024; Hao et al., 2024) in distinct anatomical and regenerative contexts.

This study had several innovative aspects. It systematically validated CSDs models in the rabbit cranium and ulna to enable the controlled evaluation of synthetic bone substitutes, while effectively excluding the influence of intrinsic bone healing. By directly comparing the properties of porous CaP ceramics with different physicochemical properties, it reveals the pivotal role of physicochemical properties in modulating bone regeneration. Additionally, it provides a comprehensive assessment of the long-term regenerative potential of CaP ceramics in CSDs, offering critical insights into their site-specific performance and clinical applicability (Zhu et al., 2023). The findings may significantly advance the understanding of synthetic bone substitutes and contribute to addressing ongoing challenges in CSDs repair.

## 2 Materials and methods

### 2.1 Physicochemical characterization of CaP ceramics

Porous TCP-B ceramic (discs, Φ12.6 × 4.0 mm) and porous biphasic calcium phosphate ceramic (BCP, discs, Φ12.6 × 4.0 mm, and cylinders, Φ5.0 × 15.0 mm) were provided by Kuros Biosciences BV (Bilthoven, Netherlands) in a sterile manner (gamma-irradiated at 25 KGy). The materials were characterized with X-ray diffraction (XRD; MiniFlex II, Rigaku, Tokyo, Japan) to determine their chemical compositions, mercury porosimetry (AutoPore IV 9500, Micromeritics) to analyze their strut pore-size distributions, scanning electron microscopy (SEM; XL30, ESEM-FEG, Philips, Eindhoven, Netherlands) to obtain their surface topography, and stereomicroscopy (Nikon, C-PS, Japan) to determine their macroporous structures. The area percentage of ceramics (M0%) was determined with histological sections (following the method described in Sections 2.5.2, 2.5.3).

### 2.2 Animals

A total of 36 New Zealand white rabbits (6 months old, 4.0–4.5 kg, Hunan SJA Laboratory Animal Co., Ltd.) were used in this study. The animals were housed in a specific pathogen-free (SPF) environment under controlled temperature conditions, with a standard diet and free access to water. Prior to surgery, the rabbits were individually housed in metal cages for at least 1 week to adapt to the new living environment.

### 2.3 Surgical operations

General anesthesia with 3% sodium pentobarbital (30 mg/kg body weight, Merck) was administered to rabbits by intravenous injection in the marginal ear vein. Surgical operations were performed under general sterile conditions following the standard protocols (Aorigin, Chengdu, China). Penicillin (100 mg/kg, China) was administered intramuscularly for 3 consecutive days post-operation to prevent infection.

#### 2.3.1 Cranium

**FIGURE 1:**
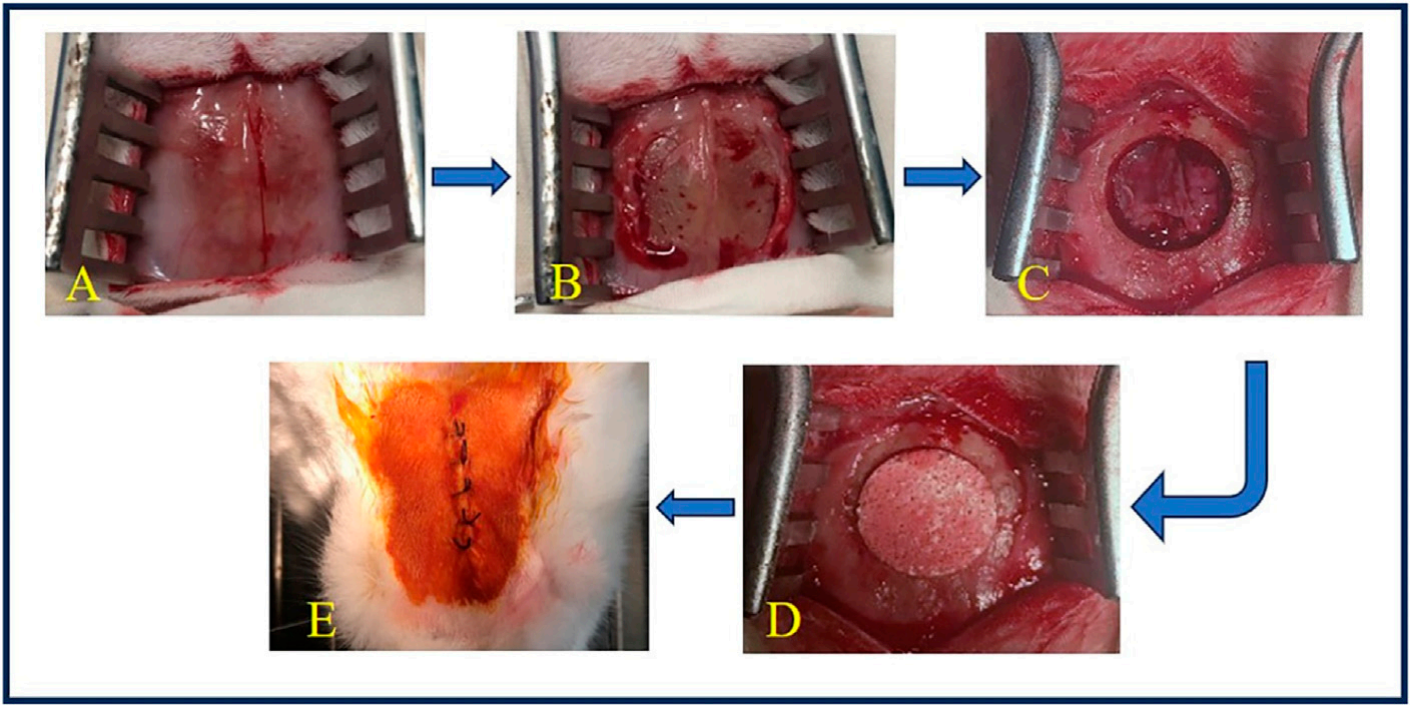
Surgery of cranial implantation. **(A)** The skin was incised to expose the surgical field. **(B)** The periosteum was detached to reveal the bone surface. **(C)** A circular bone defect with a diameter of 12.6 mm was created using a high-speed drill. **(D)** TCP-B or BCP was implanted into the bone defect. **(E)** The wound was closed in layers with silk sutures and disinfected with iodine.

Rabbits were placed in a prone position, and a midline cranial incision was made in the periosteum (Figure 1A). The overlying periosteum was then removed to expose both parietal bones (Figure 1B). A bone defect was then created with a high-speed drill (Φ12.6 mm) on the cranium at 12,000–15,000 rpm with copious saline irrigation. During the osteotomy, precautions were taken to avoid injuring the dura mater under the bone. The circular bicortical bone segment was subsequently mobilized and luxated using a thin osteotome. The defect was filled with Φ12.6 × 4.0-mm ceramic discs through press-fitting (Figure 1D) or left as empty (sham control, Figure 1C). The wounds were finally sealed with 5–0 silk sutures layer by layer and sterilized with iodine (Figure 1E).

#### 2.3.2 Ulna

**FIGURE 2:**
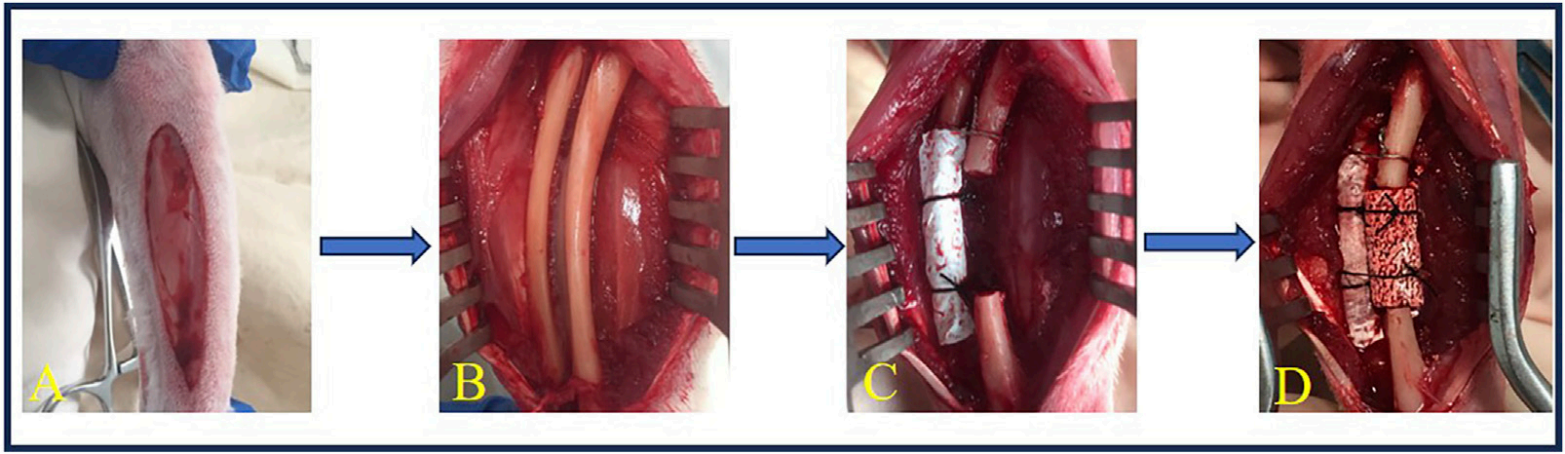
Surgery of ulna implantation. **(A)** A longitudinal incision was made on the forelimb of the rabbit. **(B)** The muscle was dissected to expose the ulna and radius, and the periosteum was elevated from the ulna and radius. **(C)** A 15.0-mm ulna defect was created using a high-speed burr, followed by ePTFE membrane coverage of the radius, secured with 6–0 silk sutures. **(D)** BCP cylinders (ø5.0 × 15.0 mm) were loaded in the defects and fixed with 6–0 silk sutures.

Rabbits were placed in a lateral position, and a longitudinal skin incision was made on the forelimb (Figure 2A). The muscle was separated to expose the ulna and radius. The periosteum was then removed from both bones (Figure 2B). An ulna segment (<15.0 mm in length) was removed with a high-speed rotary burr (M type, ø2.35 mm, China) at 12,000–15,000 rpm, and a 15.0-mm defect was achieved with a high-speed rotary burr (A type, ø2.35 mm, China), along with copious saline irrigation. The radius was then covered with an ePTFE membrane (25.0 × 19.0 × 0.1 mm, Aorigin, China) and fixed with 4–0 silk sutures, and the proximal end of the ulna was fixed to the radius with a stainless wire (ø0.5 mm) (Figure 2C). BCP cylinders (ø5.0 × 15.0 mm) were loaded in the defects and fixed with 4–0 silk sutures (Figure 2D) or left empty (sham control, Figure 2C). The wounds were finally sealed with 4–0 silk sutures and sterilized with iodine. The operated leg was supported with an aluminum support-brace finger splint (90 × 22 mm) and covered with a medical gauze for 3 days.

### 2.4 Sample harvesting

At predetermined timepoints, rabbits were euthanized with carbon dioxide. Gross observation was performed with respect to inflammation, infection, and distortion. Implants with surrounding tissues (e.g., bone or soft tissues) were collected and fixed in 4% paraformaldehyde (Servicebio, China) for at least 4 days with one refreshment of the fixative.

### 2.5 Sample evaluation

#### 2.5.1 Radiological analysis

After being fixed in 4% paraformaldehyde (Servicebio, China) for at least 4 days, the samples were rinsed with flowing tap water for 2 h and dried with tissues prior to X-ray radiography (DR, SZ-9, Huarunwandong, China), which was executed at a voltage of 50 Kvp and a current of 100 mA.

#### 2.5.2 Histological evaluation

After radiographic examination, the samples were rinsed with deionized water, dehydrated in gradient alcohol, and then embedded in polymethyl methacrylate (PMMA, CoolSet A, Aorigin, China) for hard tissue sections (10–20 µm) using a diamond histological saw (Aorigin, China); they were subsequently stained with 1% methylene blue (Sigma-Aldrich, St, Louis, MO) and 0.3% basic fuchsin (Sigma-Aldrich) for light-microscopy observation. Coronal sections were made for cranial implants, and sagittal sections were made for ulna implants.

#### 2.5.3 Histomorphometry

All the stained sections were digitalized using a scanner (Dimage Scan Elite 5400 II, Konica Minolta, Tokyo, Japan) to obtain overview images for histomorphometry. The percentage of bone in available space and the percentage of CaP ceramic left in the implants were quantified. In brief, the area with the ceramic material in the histological overview was selected in Image-Pro Plus 6.0 software as a region of interest (ROI), and the area was read in pixels (ROI). The CaP ceramic in ROI was subsequently pseudo-colored, and the area was read in pixels (M). Similarly, the bone in ROI was pseudo-colored, and the area was read in pixels (B). The area percentage of bone in available space (B%) was calculated as B% = B*100/(ROI-M), and the area percentage of CaP ceramic (M%) was calculated as Mt% = M*100/ROI. The resorption rate of CaP ceramic with time (R%) was calculated as R% = (Mt%-M0%) *100/M0%. Three sections per sample were subjected to histomorphometry, and the average of the three quantifications was assigned to the sample for further analysis.

### 2.6 Statistical analysis

Student’s *t*-tests, one-way analysis of variance (ANOVA) with Tukey’s post-test multiple comparisons, and two-way ANOVA with multiple Bonferroni’s post-test comparisons were performed. All the data were represented as means ± SD, and *p* < 0.05 was considered to indicate statistically significant differences.

## 3 Results

### 3.1 Physicochemical properties of the CaP ceramics

**FIGURE 3:**
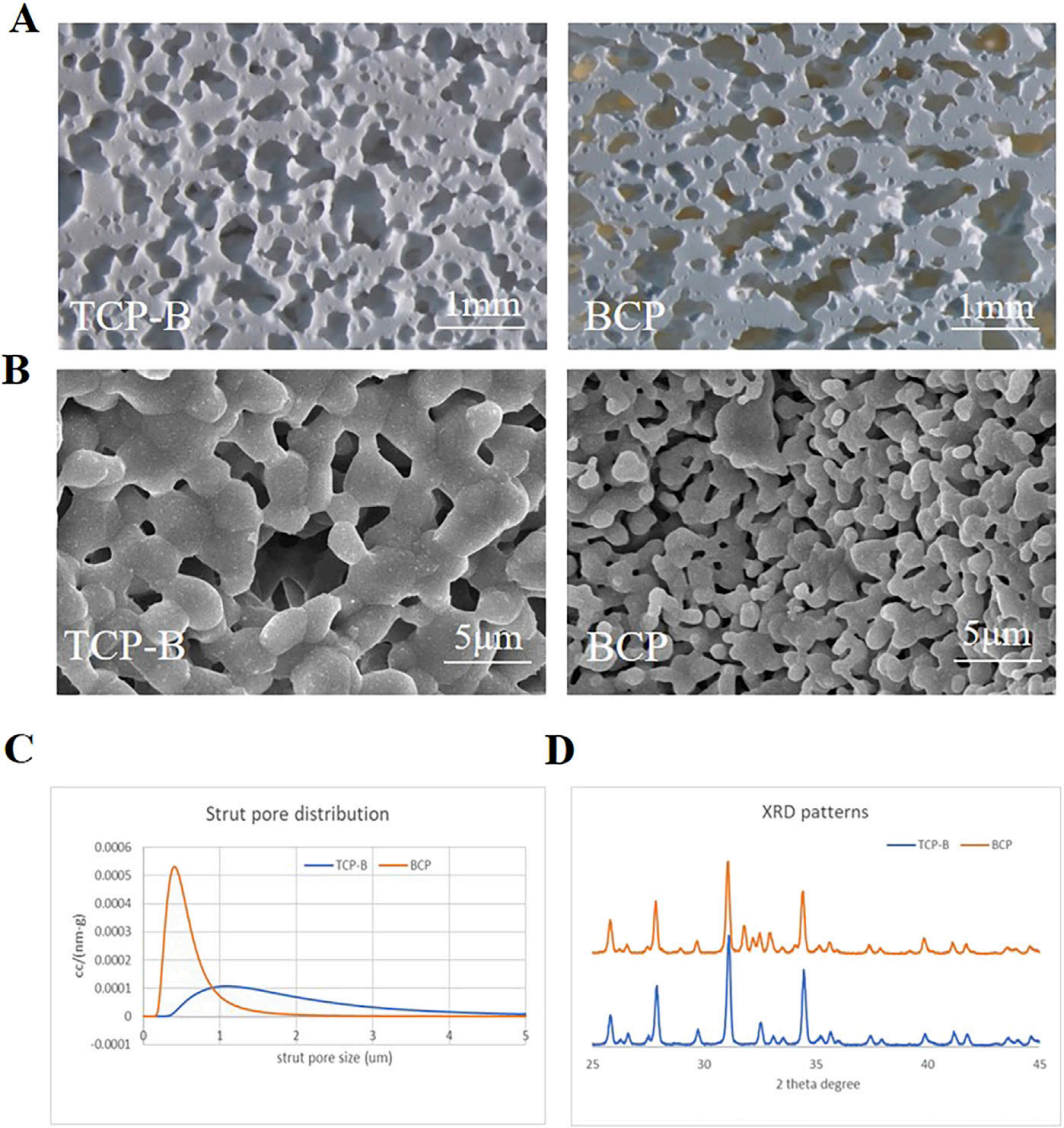
Physicochemical characteristics of materials used. **(A)** Scanning tunneling microscopy (STM) images of BCP and TCP-B; scale bars = 1 mm. **(B)** Surface morphology characterized by scanning electron microscopy (SEM); scale bars = 5 μm. **(C)** Strut pore size distribution of TCP-B and BCP. **(D)** X-ray diffraction.

The two CaP ceramics used in the study had similar macroporous structures (Figure 3A) but different surface structures, with larger crystal grains and larger surface pores being found in TCP-B than in BCP (Figure 3B). Moreover, the macroporosities of the two CaP ceramics were equivalent, with the area percentage of ceramics being 49.3% ± 3.3% for TCP-B and 45.4% ± 1.9% for BCP (*p* = 0.06). Strut pore distributions showed submicron strut pores in BCP and micron strut pores in TCP-B (Figure 3C). Chemically, BCP contained 20 ± 5%HA/80% ± 5% TCP (according to a calibration line), and TCP-B had pure β-TCP (Figure 3D).

### 3.2 Animals in study

The wounds healed without infection. Three of the 18 animals in ulna group broke their legs within 2 weeks post-operation because of their vigorous exercise and were euthanized (and thus also excluded from the final analysis). All other animals remained healthy throughout the experiments, without any complications.

### 3.3 Bone regeneration in cranial defects

#### 3.3.1 Bone regeneration in sham cranial defects at week 12

**FIGURE 4:**
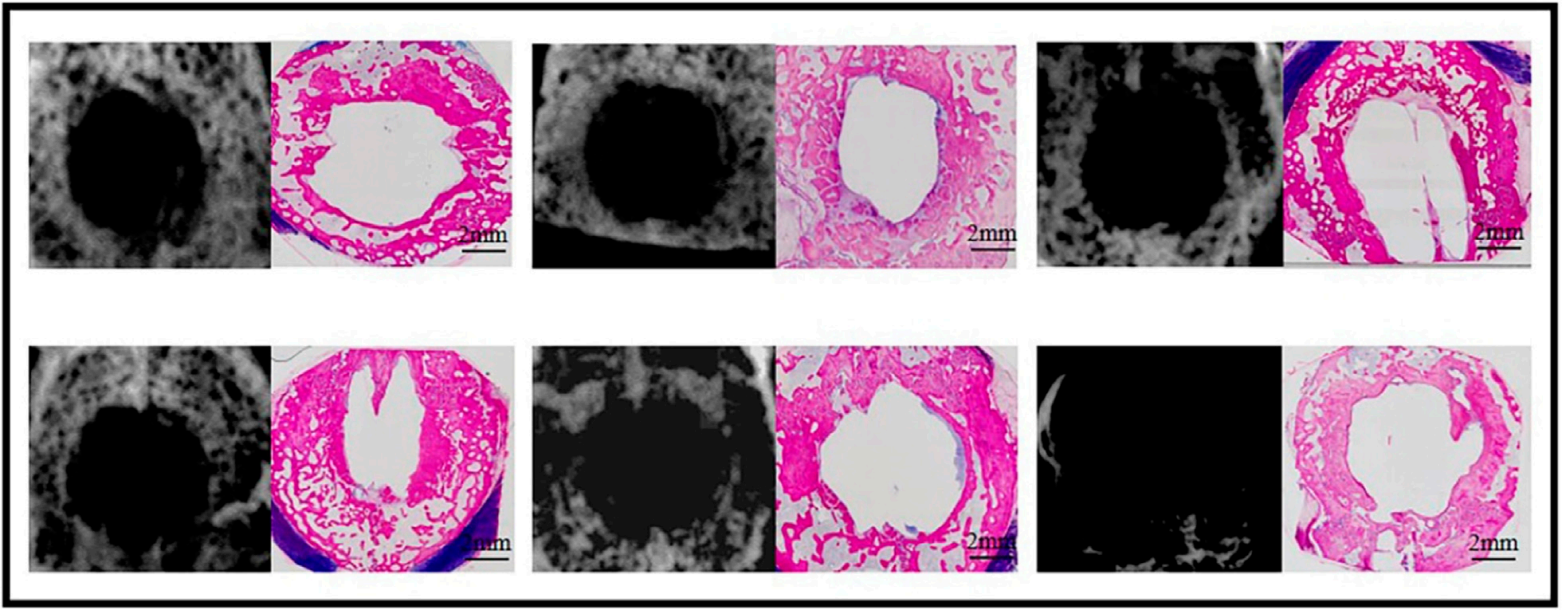
Bone formation at week 12 in the sham defects of cranial defects (X-ray and histological overviews).

Healing of the 12.6-mm cranial bone defects was not observed in any of the six samples on X-ray examination 12 weeks post-operation (Figure 4). Although bone formation was histologically observed surrounding the defects, no bone was formed in the center of any samples (n = 6) (Figure 4).

#### 3.3.2 Bone regeneration with CaP ceramics in cranial defects at week 12

**FIGURE 5:**
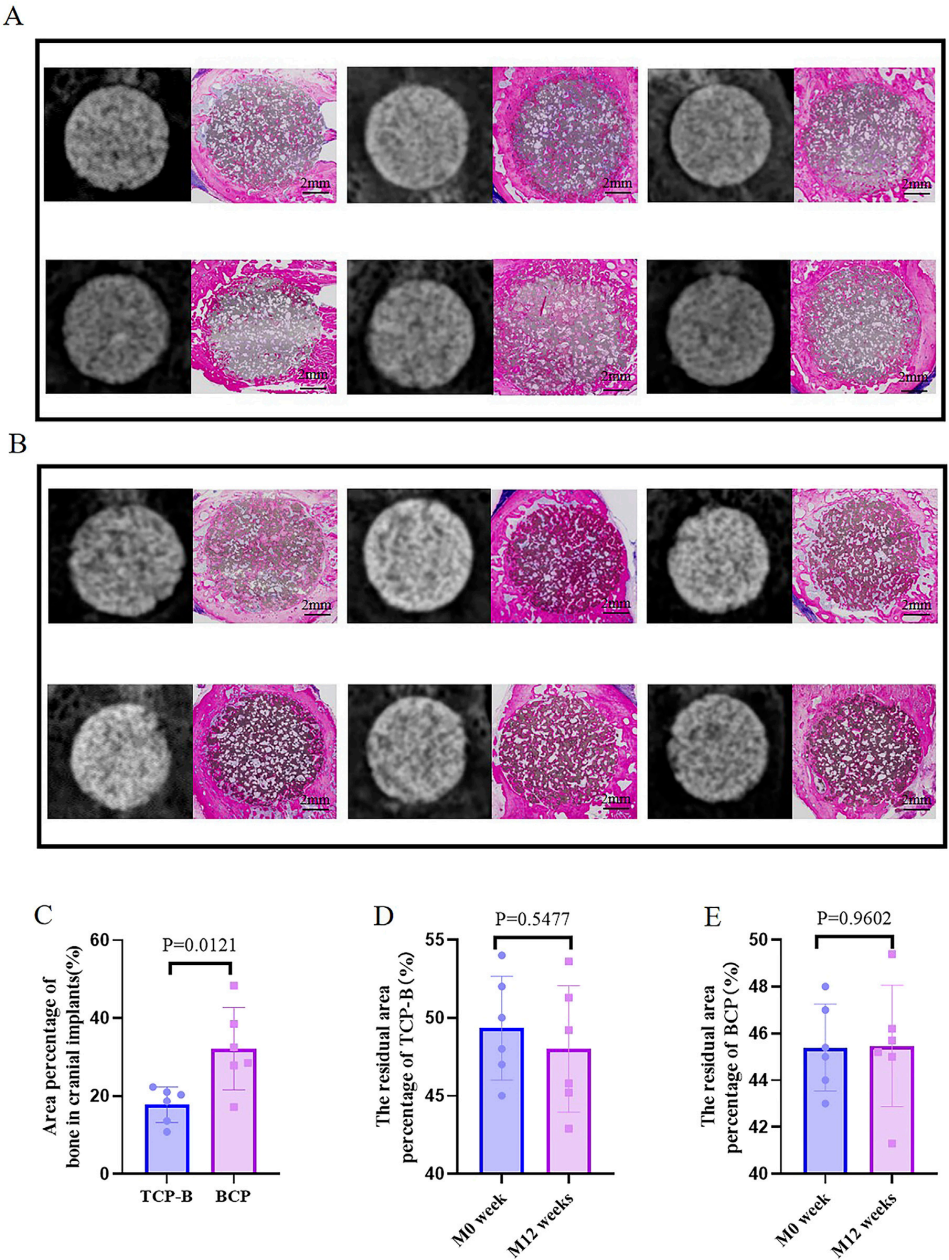
Bone formation in CaP materials at week 12. **(A)** Radiological and histological images of TCP-B. **(B)** Radiological and histological images of BCP. **(C)** Area percentage of bone in TCP-B and BCP implants in cranial defects at week 12. **(D)** Residual area percentage of TCP-B implants at different time points. **(E)** Residual area percentage of BCP implants at different time points.

Because of the presence of CaP ceramics in the cranial defects, bone regeneration in cranial defects with CaP ceramics could not be identified, and the difference between TCP-B and BCP could not be distinguished with X-rays (Figure 5). However, bone regeneration in TCP-B and BCP was different in histological overviews (Figure 5). Bone formation in TCP-B implants was less and not homogenous, showing clearly less bone in the central regions of the implants (Figure 5A). More voluminous and homogenous bone could be observed throughout the BCP implants at week 12 (Figure 5B). Increased bone formation in BCP than in TCP-B implants was also confirmed with the area percentage of bone in the available space, with 17.8% ± 4.6% of the bone being in space available for TCP-B and 32.2% ± 10.6% being in space available for BCP (*p* = 0.0121) (Figure 5C). A decrease in the ceramic percentage in the 12-week implantation was not observed for either ceramic. At week 12, the area percentage of ceramic in TCP-B implants was 48.0% ± 4.1% (compared with M0% = 49.3 ± 3.3% and *p* = 0.5477) (Figure 5D), while it was 45.5% ± 2.6% in BCP implants (compared with M0 = 45.4 ± 1.9% and *p* = 0.9602) (Figure 5E).

### 3.4 Bone regeneration in ulna defects

#### 3.4.1 Bone regeneration in sham ulna defects at week 24

**FIGURE 6:**
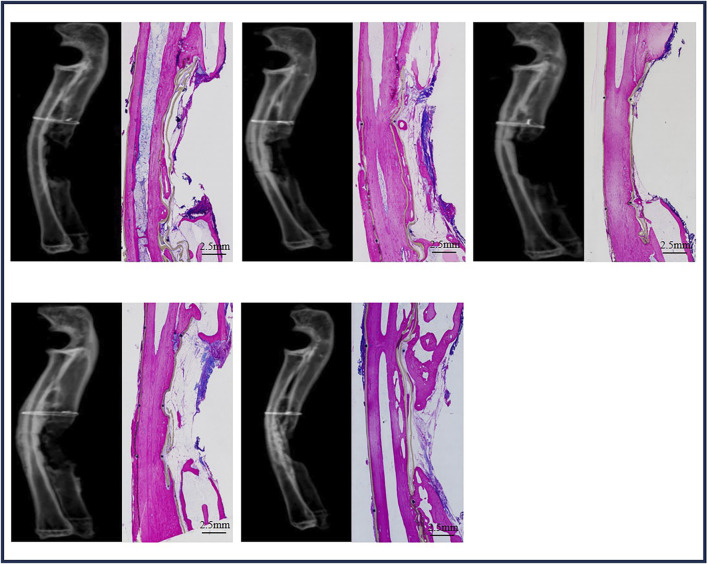
Bone formation in ulna sham at week 24.

Five sham ulna samples were used for evaluations at week 24 (Figure 6). Union of bone was not observed in any of the five samples with X-rays, and this was confirmed through histological overviews. Bone grew from both ends of the ulna defects, but a bone bridge was not formed in any of the five sham ulna samples.

#### 3.4.2 Bone regeneration in ulna defects with BCP at week 12

**FIGURE 7:**
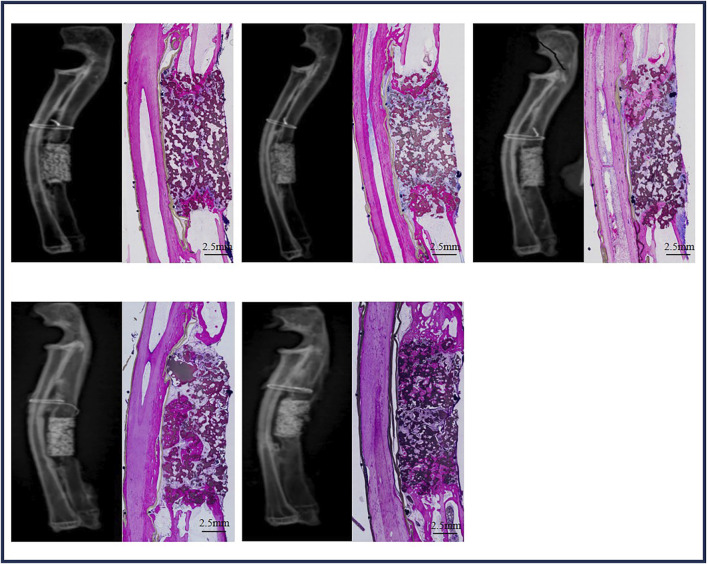
Bone formation in ulna defects with BCP at week 12.

Five ulna samples with BCP were used for evaluations at week 12 (Figure 7). Bone formation in BCP could not be confirmed via X-rays. However, histological overviews showed conductive bone formation extending from both ends of the ulna defects. Although a bone bridge had not been achieved as yet, sporadic bone formation could be observed in the central region of the defects. At week 12, less bone formation was observed, with 19.3% ± 7.3% of the available space being filled with new bone. A slight but significant decrease in ceramic was observed in the 12-week implantation, while 14.8% of BCP ceramic was resorbed in 12 weeks (from M0% = 45.4 ± 1.9% to M12W% = 38.7 ± 3.6% and *p* = 0.0379) (Figure 9B).

#### 3.4.3 Bone regeneration in ulna defects with BCP at week 24

**FIGURE 8:**
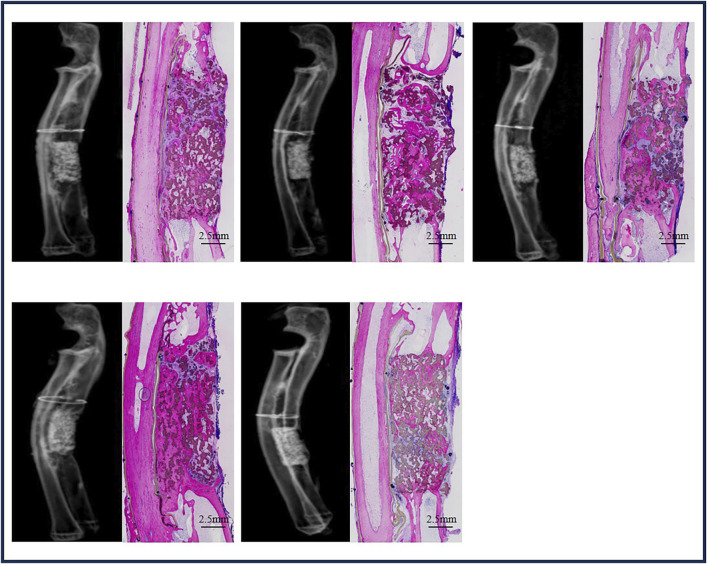
Bone formation in ulna defects with BCP at week 24.

**FIGURE 9:**
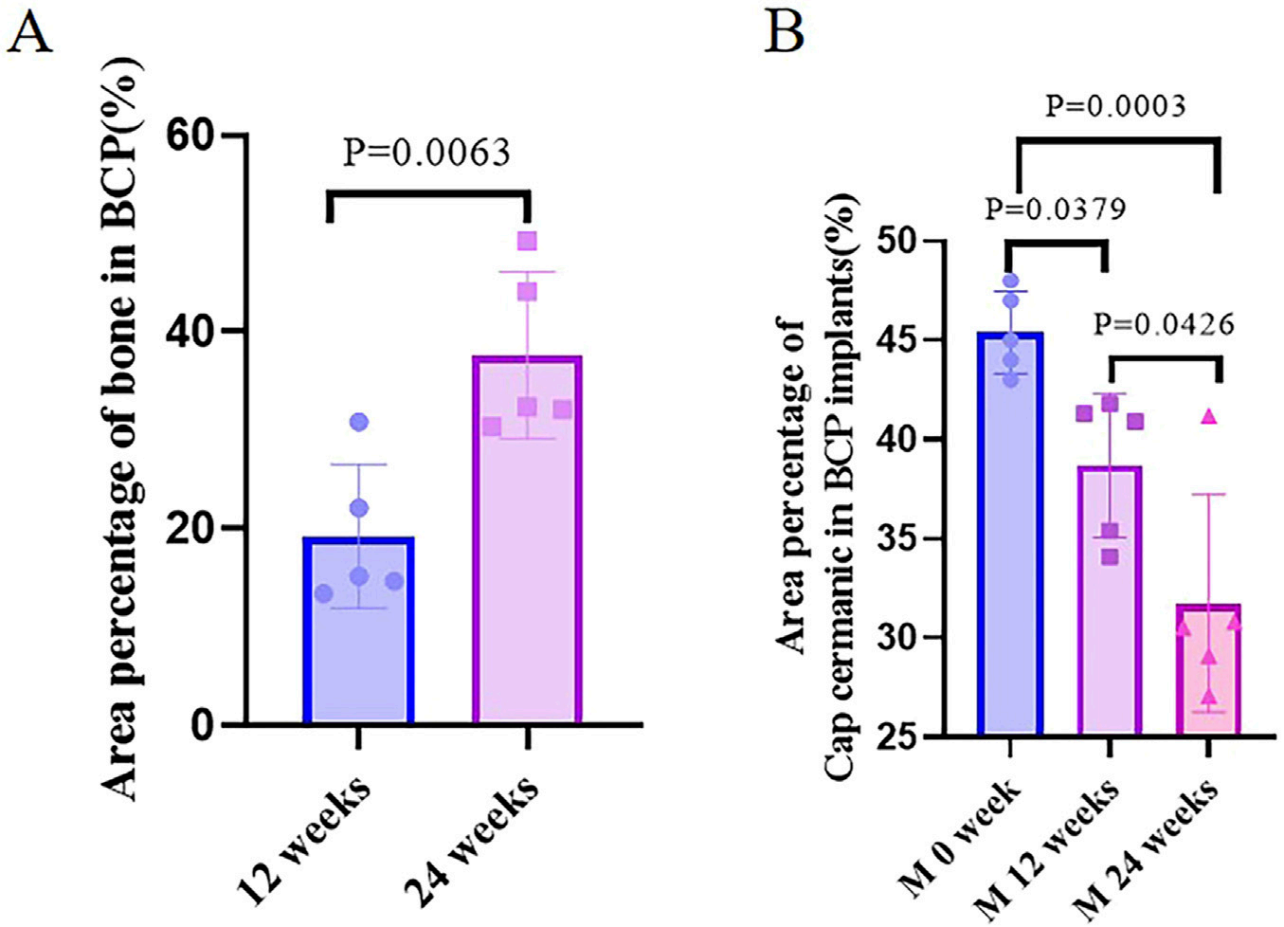
Quantification of bone. **(A)** Area percentage of bone in BCP implanted in cranial defects at weeks 12 and 24. **(B)** Area percentage of CaP ceramics in BCP implants at different time points.

Five ulna samples with BCP were used for evaluations at week 24 (Figure 8). Bone formation in BCP implants could not be confirmed with X-rays because of the strong X-ray signal of BCP. Histologically, abundant bone was formed in the whole ulna defects. Bone union appeared in four of five ulna samples with BCP implants (80% union). Quantitatively, the bone in the defects significantly increased from week 12 to week 24 (19.3% ± 7.3% at week 12% vs. 37.7% ± 8.5% at week 24, *p* = 0.0063) (Figure 9A). Additionally, the area percentage of BCP ceramic in the implants decreased further from week 12 to week 24, with 31.7% ± 5.5% ceramic being leftover at week 24 (compared with M0% = 45.4 ± 1.9%, *p* = 0.0003 and compared with M12W% = 38.7 ± 3.6%, *p* = 0.0426), meaning that 30.2% of the BCP ceramic was resorbed in 24 weeks (Figure 9B).

## 4 Discussion

As a result of the bone-forming ability of bone (which is one reason that autologous bone is considered the gold standard) (Street et al., 2017), care must be taken with orthopedic models when evaluating or comparing the bone-regeneration capacity of bone substitutes. Ideally, CSDs are necessary for such purposes (Brunello et al., 2020). Although several CSDs have been reported in the literature (Wei et al., 2024), further efforts may still be needed to minimize the influence of other general factors on bone regeneration when applying CSDs in practice. By using older animals (≥6 months) to minimize the influence of age on bone regeneration (Hirata et al., 2022), removing the surrounding periosteum to exclude bone formation by the periosteum in the bone defects (Jeyaraman et al., 2021), and further blocking the bone formation from the surrounding bone damage with the ePTFE membrane (in case of ulna bone defects) (El Backly et al., 2014), we successfully built a rabbit critical-size cranial bone-defect model (ø12.6 mm at 12 weeks) and a rabbit critical-size ulna bone-defect model (15.0 mm in length at 24 weeks), as evidenced by the non-union apparent in X-ray images and the histological overviews.

When two porous CaP ceramics (TCP-B vs. BCP) were compared in the rabbit critical-size cranial defects, significantly more bone was formed in BCP implants than in TCP-B implants in 12 weeks (Figure 5C). A full repair of the rabbit critical-size cranial bone defects was achieved with BCP in 12 weeks, as evidenced by the homogenous bone formation throughout the BCP implants (Figure 5A), while the bone formation in TCP-B implants was not homogeneous, with the absence of bone in the central region being observed in the majority of TCP-B implants (Figure 5B).

The superior performance of BCP in the rabbit critical-size cranial bone defects compared with TCP-B reveals that BCP enhanced the bone-forming ability. The biological mechanism underlying the difference between BCP and TCP-B is not fully known. The osteogenic potential of biomaterials is linked to their physicochemical properties. Enhancing the hydrophilicity and surface roughness of biomaterials has been demonstrated to promote the adhesion and proliferation of osteoblasts and mesenchymal stem cells (MSCs) (Wang et al., 2016). Similarly, the incorporation of polyethylene glycolated polyglycerol sebacate into CaP ceramic scaffolds has been found to significantly improve their mechanical properties and bioactivity, enhancing the osteogenic differentiation of stem cells and facilitating bone regeneration (Ma et al., 2016). The improved bone-forming ability of BCP could be attributed to its chemistry as TCP-B and BCP were chemically different in this study, and chemical compositions appeared to influence bone formation in CaP ceramics (Tang et al., 2018). Additionally, the improved bone-forming ability could be attributed to its submicron surface topography as the dimension of surface topography could be an osteogenic factor in CaP ceramics (Zhang et al., 2014). Previous studies have indicated that calcium phosphate ceramics with submicron surface topography can induce macrophage polarization toward the M2 phenotype (Li et al., 2020), promote osteoclastogenesis (Davison et al., 2014), and subsequently secrete factors that enhance the osteogenic differentiation of stem cells, thereby promoting bone formation (Li D. et al., 2023; Li M. et al., 2023). Surface topography appeared to be superior to chemistry in controlling bone formation in CaP ceramics (Müller et al., 2008; Duan et al., 2017), so it is likely that, in addition to chemistry, the submicron surface topography might have played a role in improving the bone-forming ability of BCP in the current study, although the mechanism is still not fully understood.

With its improved bone-forming ability, BCP could repair rabbit critical-size ulna defects. Although bone formation in ulna BCP implants was limited at 12 weeks, bone formation increased significantly with time, and abundant bone was formed in ulna BCP implants at week 24, resulting in the full repair of rabbit critical-size ulna defects with BCP at week 24 in the majority of the bone defects (4 of 5, 80% union), as shown in the histological overviews.

As shown in the current study, BCP could repair both rabbit critical-size cranial bone and ulna bone defects, while the time to repair critical-size ulna bone defects was different. A 12.6-mm critical-size cranial bone defect could be repaired in 12 weeks, but more time (e.g., 24 weeks) was needed to repair a critical-size ulna bone defect (15 mm in length). Next to the larger size of an ulna bone defect (15.0 mm in length vs. 12.6 mm in diameter), the presence of less osteogenic tissue (e.g., host bone in those cases) near the BCP ulna implants may have delayed the repair of critical-size ulna bone defects. BCP cranial implants were surrounded by host bone and accepted bone ingrowth from surrounding bone, while bone grew into BCP ulna implants from the host bone bed on the two sides of the ulna defect. As a result, significantly less bone was formed in BCP ulna implants than in BCP cranial implants at week 12 (19.3% ± 7.3% bone in BCP ulna implants and 32.2% ± 10.6% bone in BCP cranial implants, *p* = 0.0236). Twenty-four weeks to repair a bone defect may be too long in practice, while such a long time is an extreme case as the bone growth from the surrounding osteogenic tissues is maximally blocked by removing the periosteum and covering the radius next to ulna defects in this study. In a real clinical situation, the osteogenic tissues surrounding the bone defects may be preserved as much as possible. Hopefully, it will take less time (e.g., less than 24 weeks) to repair a bone defect if the surrounding osteogenic tissues (e.g., the host bone and periosteum) have been maximally preserved.

In addition to their influence on bone formation in CaP ceramic implants, the implantation sites (cranial defects vs. ulna defects) affected the resorption rates of CaP ceramics. The resorption rates of CaP ceramics generally varied with their chemistry: the higher the TCP content, the quicker the resorption (LeGeros, 1993). However, neither TCP-B (with 100% TCP) nor BCP (with 80% TCP) was resorbed in 12 weeks in cranial defects, while 14.8% BCP was resorbed in ulna defects over the same period. The enhanced resorption of CaP ceramics in ulna defects might be because of the mechanical loading, which affects the functions of osteoclasts (or other multi-nucleated giant cells) (LeGeros, 1993; Sun et al., 2023).

While orthopedic models are important in evaluating/comparing bone-regeneration capacities of bone substitutes, so are methods that analyze the outcomes for reliable conclusions. X-rays and uCT are often used to analyze bone regeneration in clinics (Schwarzenberg et al., 2020); however, as shown in the current study, bone regeneration was hardly identified due to the strong signal of CaP ceramics in X-rays (the same challenge applies to μCT), meaning that the data obtained using X-rays or μCT might not be accurate enough to draw reliable conclusions once the bone substitutes (e.g., CaP ceramics and metallic implants) have created strong signals under X-ray analysis. Although it is impossible to apply in clinics, histology (especially histological overviews) that can distinguish bone from bone substitutes and allow for the quantification of bone may be a reliable method in pre-clinical studies on bone regeneration.

This study established two critical-size bone-defect models in rabbits to evaluate and compare bone-regeneration strategies, with a particular focus on the use of various bone substitutes. These models offer flexibility for different research purposes, including examining loading versus non-loading conditions, resorption versus non-resorption, or varying rates of bone repair, with cranial defects demonstrating higher experimental success rates than ulna defects owing to the risk of limb fractures. The CSDs models proved to be effective in differentiating bone substitutes, including autografts, allografts, tissue-engineered constructs, growth factor-containing grafts, and synthetic materials. Thus, they provide a valuable platform for selecting optimal clinical strategies. Moreover, these models’ utility can be extended to evaluate non-substitution-based regeneration approaches, including millimeter-wave therapy (Jing et al., 2024) and intelligent nanosystems (Hao et al., 2024).

A particular focus of this study was on the use of porous CaP ceramics for bone regeneration in CSDs, with endpoints extending to 12 weeks or more. Future research will address early tissue responses to these ceramics and conduct *in vitro* investigations with bone-forming and innate immune cells to further elucidate the mechanistic differences between BCP and TCP-B.

## 5 Conclusion

CSDs were successfully established in the rabbit cranium and ulna. In the cranial CSDs model, BCP implants achieved significantly greater bone formation at 12 weeks than TCP-B implants. In the ulna CSDs model, bone formation and implant resorption within the BCP implants increased significantly from 12 weeks to 24 weeks, and BCP implants demonstrated a union rate of 80% at 24 weeks. These results indicate the important role of physicochemical properties in controlling bone formation in synthetic bone substitutes and indicate a strategy of physicochemical modification for synthetic materials that can be used to repair CSDs. Lastly, the CSDs models validated in the current study can be applied to evaluate bone-regeneration strategies other than bone substitution.

## Data Availability

The raw data supporting the conclusions of this article will be made available by the authors, without undue reservation.
